# Beyond clinical observations: a scoping review of AI-detectable observable cues in borderline personality disorder

**DOI:** 10.3389/fpsyt.2024.1345916

**Published:** 2024-12-10

**Authors:** Sara Močnik, Urška Smrke, Izidor Mlakar, Grega Močnik, Hojka Gregorič Kumperščak, Nejc Plohl

**Affiliations:** ^1^ Unit for Paediatric and Adolescent Psychiatry, Division of Paediatrics, University Medical Centre Maribor, Maribor, Slovenia; ^2^ Laboratory for Digital Signal Processing, Faculty of Electrical Engineering and Computer Science, University of Maribor, Maribor, Slovenia; ^3^ Department of Psychiatry, Faculty of Medicine University of Maribor, Maribor, Slovenia; ^4^ Department of Psychology, Faculty of Arts, University of Maribor, Maribor, Slovenia

**Keywords:** borderline personality disorder, review, digital biomarkers, observable cues, language use, speech, facial expression, physiological measurements

## Abstract

Borderline Personality Disorder (BPD), impacting approximately 2% of adults worldwide, presents a formidable challenge in psychiatric diagnostics. Often underdiagnosed or misdiagnosed, BPD is associated with high morbidity and mortality. This scoping review embarks on a comprehensive exploration of observable cues in BPD, encompassing language patterns, speech nuances, facial expressions, nonverbal communication, and physiological measurements. The findings unveil distinctive features within the BPD population, including language patterns emphasizing external viewpoints and future tense, specific linguistic characteristics, and unique nonverbal behaviors. Physiological measurements contribute to this exploration, shedding light on emotional responses and physiological arousal in individuals with BPD. These cues offer the potential to enhance diagnostic accuracy and complement existing diagnostic methods, enabling early identification and management in response to the urgent need for precise psychiatric care in the digital era. By serving as possible digital biomarkers, they could provide objective, accessible, and stress-reducing assessments, representing a significant leap towards improved psychiatric assessments and an invaluable contribution to the field of precision psychiatry.

## Introduction

Psychiatric conditions cause disability, premature mortality, and strain on society due to costly and prolonged treatment. While research has made strides, there remains an urgent need for faster progress in identifying and managing these disorders (1). The need for improvement in understanding and managing psychiatric disorders, while relevant to many conditions, is particularly urgent for borderline personality disorder (BPD). BPD is complex, making its diagnosis and treatment unique challenges. It affects around 2% of adults globally, and leads to adverse outcomes like education and career difficulties, shorter relationships, conflicts, risky behaviors, limited social support, reduced life satisfaction, and increased healthcare use. Individuals with BPD grapple with emotional regulation, an unstable self-concept, and relationship problems. BPD symptoms are broad and can change over a lifetime, often involving maladaptive behaviors such as occasional aggression, heightened rejection sensitivity, self-harm, and suicidal thoughts (2). In fact, individuals with BPD face an increased risk of premature death (3). Unfortunately, up to 10% succumb to suicide (4). This heightened mortality risk translates to an estimated loss of 5·0-9·3 years of life expectancy (5).

Despite several structured and semi-structured interviews, mental health diagnosis and treatment heavily depend on unstructured psychiatric interviews and subjective assessments. This reliance results in the underdetection of BPD, with over 40% of patients misdiagnosed as depressed. The complex overlap of BPD and depression symptoms underscores the critical need for precise diagnosis (6, 7). Accurate diagnosis is a crucial step for most individuals with BPD, providing significant relief and enabling them to comprehend their behaviors and past experiences. It precedes vital psychoeducation and treatment (2).

Artificial intelligence (AI) has advanced notably in healthcare, particularly in oncology, radiology, and dermatology. However, uptake of AI in psychiatry is notably slower and more challenging compared to other fields of medicine (8). This phenomenon can be attributed to several factors, such as, subjectivity of symptoms and diagnosis (1, 9), complexity of symptoms and disorders (9, 10), lack of uniform, objective biomarkers and diverse DSM-5 diagnostic criteria (11). Psychiatry’s complex data processing and clinical decision-making surpass the challenges faced in tasks like tumor identification in medical images where AI has excelled (12). Moreover, the use of AI requires access to large amounts of patient data, which often includes sensitive information shared during therapy sessions (13). The complexity of mental disorders also requires large, diverse, and high-quality datasets to train AI models effectively. However, most current datasets are small, lack diversity, and may not accurately represent the broad spectrum of psychiatric conditions (9). Thus, AI models in psychiatry often suffer from the limitation of insufficient and biased training data. AI has the potential to transform the field of psychiatry, including those working with BPD patients, however, not as a classifier generating diagnosis, but rather by providing tools that can enhance treatment efficacy and diagnostic accuracy. For instance, AI-driven tools can assist in developing personalized treatment plans by analyzing individual patient data and by predicting which treatments are most likely to be effective for individual patients, based on their unique data profiles (14).

AI has significant potential to enhance psychiatric care, but ethical and practical concerns must be addressed. Key issues include ensuring privacy and data security (15) as digital biomarkers and especially video recordings can reveal sensitive information (16, 17) and raise concerns about misuse, re-identification, and harm to the patient-therapist relationship (15, 17). Psychiatric patients may worry about stigma and discrimination, which can impact their willingness to participate in research (16), especially when their images and behaviors are recorded (17). Implementing AI methods into clinical psychiatric practice also introduces challenges, including ensuring informed consent, especially for those with cognitive impairments, and validating digital biomarkers to prevent harm from false results. Additionally, efforts must be made to reduce bias in AI models, as biased algorithms could exacerbate existing disparities in mental health care. AI’s potential to worsen health disparities due to unequal access and cultural differences, alongside data bias affecting algorithm effectiveness, adds complexity (15). Historically, mental health research has suffered from biases, and without addressing these issues, AI could reinforce existing inequities (18). Furthermore, transparency and explainability of AI-driven decisions are crucial for building trust between clinicians and patients and ensuring that AI is used as a supportive tool rather than a replacement for human. The integration of AI into clinical practice must also contend with regulatory gaps that allow unproven products to enter the market, raising safety and exploitation concerns. Addressing these issues, particularly in the context of video-based AI tools, is crucial for the ethical deployment of AI in mental health (15).

Despite these challenges, AI holds considerable potential for improving the monitoring and screening of patients. Using natural language processing could be integrated into mainstream therapies for BPD like dialectical-behavioral therapy and mentalization therapy. AI could also help patients re-author their self-narratives into more coherent sequences, promoting mentalization and insight into causal connections and self-agency (14). The most significant potential of AI in psychiatry, however, lies in the monitoring/screening of patients (19, 20). Namely, when used to extract digital biomarkers, AI is leveraged for delivering real-time insights and comprehensive contextual analysis, rather than for classifying disorders. In this area AI has demonstrated strong reliability (high accuracy and efficiency) in extracting relevant features, such as discrete and categorical data, from various datasets (21). While, when it comes to classification tasks, AI often encounters challenges related to data quality and inherent biases and explainability, which can significantly affect its performance. As such, AI can be employed to develop automated screening and assessment tools that quickly identify individuals at risk for mental health disorders (22, 23). A growing area of interest explores the connections between observable cues like language patterns, speech nuances, and facial expressions and the psychological characteristics of those who display them (24). These observable cues appear naturally, spontaneously and are less susceptible to cognitive biases and social acceptability concerns, enhancing the objectivity of psychiatric assessments (25). Digital biomarkers, using AI and observable cues such as language patterns and facial expressions, can advance the management of psychiatric disorders, including complex ones like BPD. They provide objective, accessible, and stress-reducing assessments, empowering patients and identifying high-risk individuals, aligning with precision medicine. This integration addresses the urgent need for precise psychiatric care and promises to revolutionize mental health management in the digital era (26).

This scoping review aims to inveil concealed observable features of BPD in conversations that can be harnessed by AI methods. By extensively summarizing literature results on cues like language, speech, facial expressions, nonverbal communication, and physiological measurements, our research provides valuable insights into distinct BPD characteristics. This knowledge could contribute to the development of sensing technology and machine learning algorithms, potentially supporting and refining existing psychiatric methods for earlier diagnosis and more personalized treatment of this often-overlooked disorder.

## Methods

### Overview

**Figure 1:**
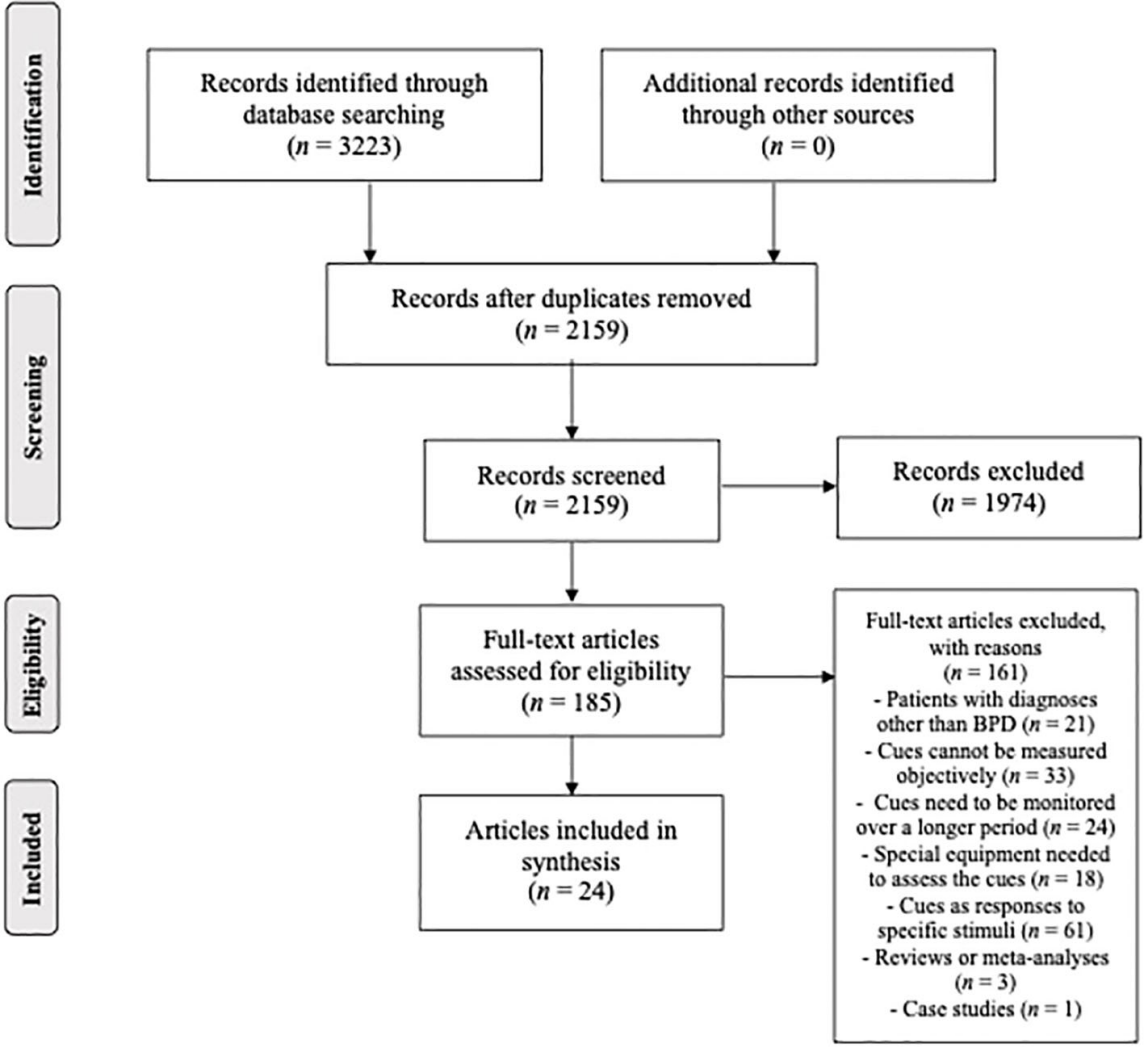
PRISMA flowchart depicting the study selection process.

A methodological framework for conducting scoping reviews by Arksey and O’Malley and Levac and colleagues was followed in the preparation of this study (27, 28). Therefore, we (1) identified the research questions, (2) identified relevant studies, (3) selected final studies to be included in the review, (4) charted the data, and (5) collated, summarized, and reported results. Preferred Reporting Items for Systematic Reviews and Meta-Analyses extension for Scoping reviews (PRISMA-ScR, see **Figure 1**) was followed to ensure that the process was systematic, complete, and transparent (29).

### Identifying the research question

Our research question aimed to identify which observable cues, spontaneously expressed by individuals with BPD, can be objectively measured during a clinical interview or in a home setting. We focused on identifying cues such as language use, speech patterns, facial expressions, other forms of nonverbal communication, and physiological measurements. To guide this investigation, we established specific inclusion and exclusion criteria, which are explained in the following section.

### Identifying relevant studies

Scopus and Web of Science, two large and extensively used databases were used to identify the relevant papers (30). These databases complement each other well (31), and are known for their large overlap with other sources, such as MEDLINE and EMBASE (32). After several rounds of preliminary searches in both databases, which helped us refine our search strategy, we carried out the main search on March 31, 2023. The final version of our search strategy combined different terms related to *borderline personality disorder* and *observable cues* (**Table 1**). All terms were searched for in their singular and plural forms, as well as British and American English. The terms were later combined into a nested format using Boolean operators (AND, OR) and searched for in titles, abstracts, and keywords. The exact search string was (“borderline personality disorder” OR “emotionally unstable personality disorder” OR “emotional intensity disorder”) AND [((sign OR cue OR express* OR feature OR indicator OR marker OR indices OR property OR reaction OR synchrony OR characteristic OR pattern] AND [text OR video OR image OR audio OR speech OR language OR paralinguistic OR prosodic OR semantic OR acoustic OR lexical OR facial OR visual OR appearance-based OR vocal OR written OR verbal OR nonverbal OR conversational OR behavioral OR behavioural OR movement OR soft)] OR [“respiratory sinus arrhythmia” OR “blood pressure” OR pulse OR “heart rate” OR “respiratory frequency” OR “respiration rate” OR “emotional recovery” OR communication OR terminology)].”

**Table 1:** The terms used in our search strategy.

Borderline personality disorder	Observable cues		
	*Words for cues*	*Way of expression*	*Additional observable cues*
“borderline personality disorder”	sign	text	“respiratory sinus arrhythmia”
“emotionally unstable personality disorder”	cue	video	“blood pressure”
“emotional intensity disorder”	express*	image	pulse
	feature	audio	“heart rate”
	indicator	speech	“respiratory frequency”
	marker	language	“respiration rate”
	indices	paralinguistic	“emotional recovery”
	property	prosodic	communication
	reaction	semantic	terminology
	synchrony	acoustic	
	characteristic	lexical	
	pattern	facial	
		visual	
		appearance-based	
		vocal	
		written	
		verbal	
		nonverbal	
		conversational	
		behavioral	
		movement	
		soft	

To be considered for inclusion, the records had to: 1) be available in English and 2) be published in scientific journals or conference proceedings. Records were then excluded if: 1) they employed samples of patients with disorders other than BPD (e.g., depression), 2) focused on cues that cannot be measured objectively (e.g., reliance on self-report), 3) focused on cues that need to be monitored over a longer period (e.g., monitoring patients for 24 hours), 4) focused on cues that need specialized equipment to be assessed (e.g., brain imaging techniques), 5) focused on cues that are responses to specific stimuli (e.g., recording eye movements during cognitively demanding tasks), 6) they were reviews or meta-analyses, and 7) if they were single case studies (i.e., with *N* = 1). The methodological quality of studies was not a reason for exclusion.

### Study selection

The main search, performed in SCOPUS and Web of Science, resulted in 3223 English language articles (SCOPUS: 1570, Web of Science: 1653) published in scientific journals and conference proceedings. An additional search in Google Scholar was also performed to ensure we didn’t miss any relevant records, but it did not lead to the identification of any previously unidentified articles. The two chosen databases overlapped considerably, which led to the removal of 1064 records. After screening the titles and abstracts, 185 records (8·6% of identified unique records) still fit the criteria. However, further assessment revealed that only 24 articles (1·1% of identified unique records) met the inclusion criteria. These articles were included in the final synthesis.

### Charting the data

Following the research question, a spreadsheet form was developed and used for the extraction of the following information from the reviewed papers: (1) authors, (2) year of publication, (3) country where the study was carried out, (4) sample characteristics, (5) BPD diagnosis, (6) (potential) psychiatric comorbidity, (7) comparison group, (8) observed cues, and (9) methods of observation. The data were extracted from the reviewed papers by two researchers (SM and (NP)) and refined in an iterative process while reviewing the papers.

### Collating, summarizing, and reporting results

We followed the aim of scoping reviews, i.e., mapping of the existing findings and providing their overview in a descriptive manner (27). The results were analyzed by three authors (SM, HGK and NP) via thematic analysis (33). Main categories of cues of borderline personality disorders were predefined, and included features related to (1) language use, (2) speech, (3) facial expressions, (4) other features of nonverbal communication, and (5) features related to physiological measurements. The process of collating and summarizing results was reviewed by two authors (NP and US).

## Results

### Characteristics of reviewed studies

While the publication year ranged from 1993 to 2022, more than 75·0% of included studies (*n* = 19; 79·2%) were published in the last ten years, and precisely 50·0% (*n* = 12) were published in the last five years. Geographically, most of the studies (*n* = 17; 70·8%) were conducted in Europe, followed by studies conducted in North America (*n* = 5; 20·8%), Australia (*n* = 1; 4·2%), and Asia (*n* = 1; 4·2%).

The reviewed studies involving human participants had sample sizes ranging from 10 to 143 (average 58·1, SD = 39·0), but BPD patients were only a subset of the sample. Additionally, the three studies without human participants analyzed from 225 to 149,798 text posts. In studies reporting demographic data, the samples were entirely or predominantly female, whereas the average age of participants was mostly (73·9%) in the 25-35 range. Most studies used DSM (either DSM-IV or DSM-V) criteria to diagnose BPD (*n* = 17; 70·8%), while others used different ways of diagnosing BPD (*n* = 2; 8·3%) or did not attempt a diagnosis (*n* = 5; 20·8%). Nearly half (47·4%) reported significant comorbidity in their samples.

Regarding observed cues, half of the studies (*n* = 12; 50·0%) focused on physiological cues like heart-rate variability, blood pressure, skin conductance, and respiratory sinus arrhythmia. The other studies primarily examined language use (*n* = 6; 31·6%), facial expressions (*n* = 2; 8·3%), speech (*n* = 1; 4·2%), and non-verbal communication (*n* = 1; 4·2%). Two studies (*n* = 2; 8·3%) observed cues from multiple categories. Various methods, including electrocardiogram, Ag/AgCl electrodes, electromyogram, videotaping, audio recording, and coding systems, were used to measure these cues objectively. Patient data were compared to healthy controls in 45·8% of studies (n = 11), other disorder patients in 12·5% (n = 3), both healthy controls and other disorder patients in 20·8% (n = 5), and in the remaining 20·8% (n = 5), there was no comparison group. For more details, refer to **Table 2**.

**Table 2:** Summary of the articles included in the review.

Authors, year, country	Sample	BPD diagnosis	Psychiatric comorbidity	Comparison group	Observed cues	Methods of observation
Aleknaviciute et al. (2016), Netherlands (34)	*N* = 81, *n*_BPD_ = 26 non-hospitalized patients (gender: 100·0% female; age: *M* = 29·2, *SD* = 6·4)	Yes (DSM-IV criteria)	Yes, some patients had co-morbid Axis II disorders	Participants with cluster C personality disorder; healthy controls	Physiological (heart’s electrical activity; skin conductance)	Electrocardiogram, Ag/AgCl electrodes
Back et al. (2022), Germany (35)	*N* = 113, *n*_BPD_ = 53 patients (gender: 100·0% female; age: *M* = 30·0, *SD* = 7·4)	Yes (DSM-IV criteria)	No information	Healthy controls	Physiological (heart-rate variability)	Electrocardiogram
Bomba et al. (2020), Italy (36)	*N* = 140, *n*_BPD_ = 70 hospitalized patients (gender: 100·0% female; age: *M* = 14·7, *SD* = 1·3)	Yes (DSM-V criteria)	No comorbidity	Healthy controls	Physiological (heart’s electrical activity)	Electrocardiogram
Bortolla et al. (2022), Italy (37)	*N* = 56, *n*_BPD_ = 28 non-hospitalized patients (gender: 100·0% female; age: *M* = 24·8, *SD* = 6·3)	Yes (DSM-V criteria)	Yes, but without acute symptomatology	Healthy controls	Physiological (heart-rate variability, respiratory sinus arrhythmia)	Electrocardiogram
Brüne et al. (2015), Germany (38)	*N* = 30, *n*_BPD_ = 15 hospitalized patients (gender: 66·7% female; age: *M* = 27·5, *SD* = 7·3)	Yes (DSM-IV criteria)	No; while some patients had depressive symptoms, they did not meet the criteria for any Axis I disorder	Healthy controls	Facial expressions (different patterns, such as affiliative behaviors)	Videotaping and coding with the ethological coding system for interviews
Carter & Grenyer (2012), Australia (39)	*N* = 24, *n*_BPD_ = 12 non-hospitalized patients (gender: 91·7% female; age: *M* = 26·6, *SD* = 9·2)	Yes (DSM-IV criteria)	Yes, some patients had co-morbid Axis I and Axis II disorders	Healthy controls	Speech (overall speech impairment, semantic complexity, syntactic complexity, pause profile)	Audiotaping, transcribing, and analyzing with various softwares
Dammann et al. (2020), Switzerland (40)	*N* = 30, *n*_BPD_ = 30 hospitalized patients (gender: 93·3% female; age: *M* = 30·8, *SD* = 6·1)	Yes (DSM-IV criteria)	Yes, some patients had co-morbid Axis I disorders and Axis II disorders	None	Facial expressions (facial affective behavior)	Videotaping and coding with the emotional facial action coding system
Dyson & Gorvin (2017), United Kingdom (41)	225 tweets	No (personal identification with BPD)	No information	None	Language use (discourse used to construct BPD)	Collecting tweets, critical discourse analysis
Ebner-Priemer et al. (2005), Germany (42)	*N* = 42, *n*_BPD_ = 21 patients (some hospitalized; gender: 100·0% female; age: *M* = 28·5, *SD* = 81)	Yes (DSM-IV criteria)	Yes, some BPD patients had co-morbid Axis I disorders	Healthy controls	Physiological (left orbicularis oculi, skin conductance, heart rate)	Electromyogram, Ag/AgCl-electrodes, electrocardiogram
Flasbeck et al. (2020), Germany (43)	*N* = 40, *n*_BPD_ = 20 patients (gender: 85·0% female; age: *M* = 31·2, *SD* = 10·4)	Yes (DSM-V criteria)	Yes, some patients had co-morbid depression, PTSD, and other disorders	Healthy controls	Physiological (heart rate-related measures)	Electrocardiogram
Geiss et al. (2021), Germany (44)	*N* = 59, *n*_BPD_ = 29 non-hospitalized patients (gender: 79·3% female; age: *M* = 39·4, *SD* = 9·8)	Yes (DSM-IV criteria)	No information	Healthy controls	Physiological (RR-intervals, blood pressure, skin conductance, respiratory frequency)	Electrocardiogram, finger-pulse photoplethysmography, Ag/AgCl electrodes, piezoelectric belt
Gemmell et al. (2019), United States (45)	149,798 text posts	No (self-declared BPD)	No information	Patients with bipolar disorder; loved ones of people with bipolar disorder or BPD	Language use (informal topics and patterns in language)	Collecting text posts, language analysis using natural language processing techniques
Grove et al. (2017), United States (46)	*N* = 143, *n*_BPD_ = 143 non-hospitalized patients (gender: 51·7% female; age: *M* = 23·2, *SD* = 5·1)	No (symptoms assessed with BSL-23)	No information	None; BPD symptoms were assessed in all participants	Physiological (blood pressure)	Vital signs monitor using the oscillometric method
Jeanneau & Armelius (1993), Sweden (47)	*N* = 30, *n*_BPD_ = 10 non-hospitalized patients	Yes (structural interview)	No information	Neurotic personality organization; psychotic personality organization	Language use (linguistic variables, such as conjunctions, negative adverbs and pronouns, and adaptor words)	Words extraction and using a text-analyzing program
Kuo et al. (2016), Canada (48)	*N* = 55, *n*_BPD_ = 25 non-hospitalized patients (gender: 64·0% female; age: *M* = 32·7, *SD* = 9·6)	Yes (DSM-IV criteria)	Yes, some patients had co-morbid bipolar, major depressive, and other disorders	Healthy controls	Physiological (heart rate, electrodermal activity, respiratory sinus arrhythmia)	BIOPAC 5-channel acquisition system
Lyons et al. (2017), United Kingdom (49)	500 forum posts (100 posted on a BPD forum)	No (self-disclosed BPD in posts)	No information	Self-disclosed generalized anxiety disorder; major depressive disorder; obsessive compulsive disorder; schizophrenia; healthy controls	Language use (emotional, cognitive, and structural components of the text)	Collecting forum posts, analysis using a linguistic inquiry and word count program
Meyer et al. (2016), Germany (50)	*N* = 91, *n*_BPD_ = 50 patients (some with current BPD, some in remission; some hospitalized; gender: 100·0% female; age: *M* = 27·9, *SD* = 5·1)	Yes (DSM-IV criteria)	Yes, some patients had co-morbid affective, anxiety, eating, and other disorders	PTSD; healthy controls	Physiological (heart-rate variability)	Electrocardiogram
Ramseyer et al. (2020), Switzerland (51)	*N* = 31, *n*_BPD_ = 16 hospitalized patients (gender: 62·5% female; age: *M* = 27·5, *SD* = 7·3)	Yes (DSM-IV criteria)	No; while some patients had depressive symptoms, they did not meet the criteria for any Axis I disorder	Healthy controls	Non-verbal communication (non-verbal synchrony)	Videotaping, automated video analyses of subject’s and interviewer’s body movement (motion energy analysis)
Sundbom & Jeanneau (1996), Sweden (52)	*N* = 25, *n*_BPD_ = 10 hospitalized patients	Yes (Structural interview)	No information	Psychotic personality organization; neurotic personality organization	Language use (various linguistic variables)	Computerized content analysis
Thomson & Beauchaine (2018), United States (53)	*N* = 104 non-hospitalized participants, some with BPD traits but no exact number reported (gender: 83·0% female; age: *M* = 19·9, *SD* = 1·2)	No (symptoms assessed with MBPD)	No information	None; BPD symptoms were assessed in all participants	Physiological (respiratory sinus arrhythmia)	Electrocardiogram, respiration belt
Villanueva-Valle et al. (2021), Mexico (54)	*N* = 10, *n*_BPD_ = 5 hospitalized patients (gender: 100·0% female; age: *M* = 28·8, *SD* = 6·4)	Yes (DSM-IV criteria)	Yes, some patients had a history of traumatic experiences and co-morbid PTSD	Healthy controls	Language use, speech, facial expressions (basic emotion expressions, emotional valence, acoustic parameters of the voice)	Videotaping, application of computational software to the visual (FaceReader) and sound (PRAAT) tracks
Walter et al. (2009), Switzerland (55)	*N* = 24, *n*_BPD_ = 12 hospitalized patients (gender: 79·2% female; age: *M* = 29·3, *SD* = 8·6)	Yes (DSM-IV criteria)	No information	Major depressive disorder	Language use (interview content)	Transcripts analyzed with computerized content analysis method
Wang et al. (2020), China (56)	*N* = 50, *n*_BPD_ = 17 non-hospitalized patients (gender: 94·1% female; age: *M* = 34·0, *SD* = 21·0)	Yes (DSM-IV criteria)	No comorbidity with bipolar disorder but no other information	Bipolar disorder; healthy controls	Language use, speech (linguistic complexity features, dependency-based propositional idea density, extraction of various speech-related variables, semantic content features, and dialogue features)	Audio recordings, transcriptions, various segmentations, evaluation using leave-one-participant-out method
Weise et al. (2020), Germany (57)	*N* = 43, *n*_BPD_ = 43 non-hospitalized patients (gender: 95·4% female; age: *M* = 15·5)	Yes (DSM-IV criteria)	No information	None	Physiological (heart-rate variability)	Electrocardiogram

### Features related to Language use

Patients with BPD within borderline personality organization (BPO) tend to favor language that highlights external viewpoints and maintains an impersonal tone. This language conveys ideas neutrally, avoiding personal attribution. Multiple studies support the prevalence of third person singular pronoun use among patients with BPD (47, 49). Notably, Jeanneau and Armelius highlight the prominence of the pronoun “they” as characteristic of BPD (47). Singular pronoun use, including “you” is heightened in BPD (49). Another distinct feature is the frequent use of future tense (47, 52).

Numerous other formal grammatical features are distinct in BPD, including adaptor words (like “so”), time-space adverbs (like “then”), conjunctions (such as “because”), intent expressions (like “I mean”), capability expressions (“I can”), negative verbs, nouns, and adjectives (like “awful”, “disgusting”, “unhappy”, “crazy”), as well as frequent negation (“not”) (47, 52). Another observed linguistic feature is the combination of nonfluencies with conjunctions. A preference for absolute words, coupled with common adverbs and article words (such as “a”, “an”, and “the”), is also noticeable. Conjunctions combined with nonfluencies, and the pairing of “you” with verbs and nonfluencies, also emerge as notable markers (56). While patients with BPD use adjectives frequently, interjections are less common (54).

When comparing bipolar disorder (BD) and BPD, several distinctions encompassing quantitative measures like Brunet’s index (BI), moving average type-to-token ratio (MATTR), and mean length sentence (MLS) emerged in Wang et al., but they do not clarify further on the actual differences between both conditions. Other notable differences include “we” with prepositions and a mix of absolute words, common adverbs, and negations. Thematic content focuses on social processes and drives. The study emphasizes the significance of linguistic complexity in differentiating BPD from BD and controls. Linguistic features play a key role, while content features have limited impact on classification. Findings underscore linguistic features’ importance, the drawback of omitting linguistic and dialogue aspects, and the relative dispensability of dialogue and content features (56).

Dyson and Gorvin unveiled discourse dynamics about BPD, depicting a spectrum from BPD as a source of tension to its depiction as a distinct and unique existence. Each of these narrative repertoires exhibited its own set of linguistic patterns and frequently employed words and phrases that actively contributed to shaping these portrayals. The “BPD as a source of tension” repertoire highlights several preferred themes, encompassing struggles for control, reductionist and deterministic terminology, expressions of powerlessness, and a biomedical standpoint. Specific linguistic patterns and commonly used words/phrases, like “disorder”, “do everything right”, “disorder takes over”, “out of nowhere disorder takes over”, “stupid things”, etc. reinforce this view. In contrast, the “BPD as a different existence” repertoire emphasizes themes of acceptance, positive self-comparisons, and openness. Using linguistic strategies describing BPD as just one of many ways to engage with the world, and the consistent use of words and expressions like “passion”, “specialness”, “embrace”, “unstable”, “abnormal”, and “different”, a coherent alternative narrative emerged (41).

In BPO language use, words often carry aggressive and depressive connotations (52). Individuals with BPD show limited negativity in interviews (55). On the other hand, their writing contains numerous negative emotion words (49). This is consistent with heightened negative emotional valence (45). Further exploration of their online conversations revealed distinctive characteristics, including an emphasis on mood-related vocabulary, a distinct focus on dating experiences (both positive and negative), a notable intertwining of work-related concerns with discussions about medication and diagnoses, and an overall optimistic outlook regarding the efficacy of treatment through medication, despite acknowledged challenges (54).

No significant differences were found between patients with BPD and healthy controls in terms of the complexity of sentence structure, or the complexity of the content being communicated (39).

### Features related to speech

Although there were no significant overall speech differences between BPD patients and healthy controls, individuals with BPD displayed notably higher pause frequency during neutral speech conditions (39). Additionally, a distinctive dialogue-related aspect emerges, encompassing number of words per second, pause duration, and relative floor control (56). Compared to the control group, patients show more frequent correlations, especially positive ones, between elements of prosody and facial expressions of certain negative emotions. Specifically, patients exhibited a positive correlation between facial expressions of disgust and anger and the acoustical parameters of adjectives and interjections, both in terms of decibels and fundamental frequency, which was absent in controls. Additionally, some correlations exhibit opposite patterns: negative in controls and positive in patients, notably for disgust and adjectives (f0-dB), anger and adjectives (dB), and anger and interjections (f0). Vocal characteristics of adjectives and interjections showed no significant differences between patients with BPD and controls (54).

### Features related to facial expressions

In contrast to healthy controls, individuals with BPD exhibited reduced affiliative behavior, concerning patterns of behavior that invite and positively reassure social interaction, including “head to side” movements; “bob”, a sharp upward movement of the head, similar to an inverted nod; “flash”, a quick raising and lowering of the eyebrows; “raise”, a movement in which the eyebrows are raised and kept up for some time; and “smile”, in which the lip corners are typically drawn back and up. Both groups demonstrated comparable inclinations to engage in flight behaviors, expressing the avoidance of social interaction and comprising behavioral features that lead to cutting off communication (38).

Regarding facial affective behavior, individuals with BPD demonstrated prominent negative emotions like disgust and contempt. However, they also exhibited social smiles, indicative of positive social interactions (40). Patients with BPD displayed less than half the amount of sadness compared to the control group, indicating a complex blend of emotional expressions (54). Cluster analysis divided patients into two groups: Cluster 1 showed higher overall facial activity and intense negative emotions (anger, contempt, disgust), combined with significant social smiles; Cluster 2 displayed lower levels of specific negative emotions while maintaining notable social smiles (40).

### Other features of nonverbal communication

Nonverbal synchrony between patients with BPD and interviewers was significantly higher than chance (pseudosynchrony). Patients with BPD often led in nonverbal interactions, with interviewers mirroring their cues more than the opposite (51).

### Features related to physiological measurements

Several studies found no significant heart rate differences between individuals with BPD and control groups at baseline (37, 42, 50). On the contrary, some studies revealed patients with BPD having elevated rates (37, 48, 57).

In terms of ECG measurements encompassing various parameters such as respiratory sinus arrhythmia (RSA), square root of the mean squared differences of successive NN intervals (RMSSD), standard deviation of normal-to-normal intervals (SDNN), and vagal-mediated heart rate variability (HRV), Bortolla et al. found no significant differences in baseline measurements between individuals with BPD and control groups (37). Likewise, no substantial differences in ECG amplitudes were observed between patients with BPD and healthy controls (43). Patients with BPD exhibited reduced RSA levels with a detected negative correlation between BPD symptom severity and RSA, indicating decreased RSA as symptoms intensify (48, 53). Females with BPD demonstrated lower mean RMSSD values compared to healthy controls (35). Regarding HRV, heightened BPD symptom severity was linked to reduced HRV during rest (57). In another study individuals with BPD showed notable heart rate fluctuations compared to other groups, without significant distinctions in HRV measures (50). The study by Flasbeck et al. unveiled significant connections among cardiovascular measures, symptom severity, and the parasympathetic nervous system (PNS) index (43). Additionally, individuals with BPD displayed longer QTcd duration than controls, aligning with findings from Bomba et al. showing a mild yet statistically significant positive correlation between QTc and QTcd measurements within the BPD group (36).

Patients with BPD showed higher systolic blood pressure (BPsys) than controls, particularly during rest. Additionally, patients with BPD consistently displayed elevated diastolic blood pressure (BPdia) in comparison to controls (44). Notably, BPD symptomatology did not predict cardiovascular reactivity (CVR), computed from BPsys and BPdia values (46).

Resting skin conductance levels (SCL) in patients with BPD resembled those of healthy controls (42). Yet Aleknaviciute et al. revealed heightened overall SCL in patients with BPD, indicating intensified physiological responses associated with emotional experiences (34).

Patients with BPD showed significantly lower mean resting scores in left orbicularis oculi EMG compared to controls (42).

## Discussion

Our study revealed numerous unique characteristics of BPD in language, nonverbal cues, and physiology. These findings provide a comprehensive understanding of BPD with potential implications for diagnosis and treatment.

Language patterns in BPD patients offer insights into their emotional regulation and cognitive processes. They often use impersonal language, including third-person pronouns like “they”, possibly linked to insecure attachments rooted in early life traumas (47, 49). Their increased use of future tense may stem from heightened uncertainty and anxiety related to emotional fluctuations and a desire for change, possibly even as an unconscious way of navigating the future (47). Unique linguistic characteristics, including vague adaptor words, adverbs, conjunctions, and negation, provide insights into coping with emotional difficulties, self-reflection, and identity integration (47). Discourse dynamics in the study uncovers two distinct narratives surrounding BPD. They often portray BPD as an existence of tension, allowing them to distance themselves from personal agency over their behaviors and the stigma attached to the diagnosis. On the other hand, some individuals construct BPD as a different existence, embracing their uniqueness and challenging prevailing conceptualizations. This narrative offers more positive subject positions and may alleviate some of the negativity associated with BPD. However, it can also lead to unintended comparisons with others, potentially hindering access to care and support (41). Concealment of intense negative emotions in interviews, in contrast to open expression in writing, reflects their greater comfort with written self-expression. This divergence in communication styles may pose interpersonal challenges, as suppressed spoken emotions hinder effective communication. Identity issues in BPD can contribute to this struggle, leading to primitive defense mechanisms like splitting that manifest in non-verbal behaviors (55).

Analyzing nonverbal behavior in BPD patients during clinical interactions offers valuable insights into their emotions, feelings, and the therapeutic relationship quality, surpassing verbal communication analysis alone (38). Speech-related findings, notably increased pause frequency during neutral speech, distinguish BPD patients from healthy controls. These pauses may result from developmental disruptions in brain networks linked to early psychological trauma, affecting the corpus callosum’s development (39). Correlations between prosody and facial expressions, particularly related to disgust and anger, reveal a strong connection between speech and nonverbal cues, with diagnostic potential. These correlations may be tied to emotional hyperreactivity and heightened sympathetic tone, facilitating intense social interactions, even with negative emotions. These intensified emotional signals may serve as a call for help, particularly during initial clinical interviews (54).

Individuals with BPD exhibit distinct social behavior patterns, including reduced affiliative behaviors and inclinations toward avoidance of social interactions. This underscores their interpersonal challenges, emphasizing the need to address emotional regulation, coping mechanisms, and social skill development in therapy. Patients with BPD show a complex interplay of emotions across different modes of expression. In spoken interviews, they display limited negativity, possibly reflecting a more neutral emotional valence, although context and potential emotion suppression need consideration. However, in written communication, BPD patients use numerous negative emotion words, indicating heightened negative emotional valence in this modality. This contrast between spoken and written expressions highlights the nuanced nature of emotional dynamics in BPD, influenced by emotional suppression, contextual factors, and alexithymia. Negative facial expressions like disgust and contempt contribute to interpersonal challenges, fostering emotional aggression and social withdrawal. The use of social smiles may serve as a defense mechanism to limit interpersonal contact while maintaining emotional distance, illustrating the multifaceted nature of emotional regulation in individuals with BPD (40). Concealing sadness and displaying anger, disgust, and contempt may result from feeling threatened, particularly in interactions with male interviewers, given their history of trauma and abuse (45, 49, 52, 54, 55).

Patients with BPD exhibit significantly elevated nonverbal synchrony with interviewers, surpassing chance levels, indicating complex behavioral and emotional interactions. This heightened synchrony possibly reflects the intense emotional experiences and interpersonal challenges often associated with BPD, illustrating emotional contagion where their feelings influence those around them. Additionally, patients with BPD tend to display fewer prosocial behaviors and engage less in social interactions, partly driven by their negatively biased facial emotional displays. This sensitive measure of nonverbal synchrony offers insights into these subtle changes in coordinated movement (51).

Physiological measurements in individuals with BPD might provide insights into their emotional responses and physiological arousal (58–61). Some studies found no significant baseline heart rate differences between BPD and control groups (37, 42, 50), while others indicated elevated heart rates in BPD patients (37, 48, 57), suggesting varying physiological arousal. The inconsistency may be due to several factors: the inclusion of participants taking medications and those with additional anxiety disorders. These factors, especially anxiety disorders associated with hyperarousal, could affect psychophysiological measures, potentially skewing the results and making them less representative of the primary condition being studied (59). ECG measurements revealed reduced respiratory sinus arrhythmia (RSA) levels in BPD patients (48, 53), which is an index of heart rate variability mediated by the vagus nerve. Reduced basal vagal activity is considered a sign of susceptibility to negative emotional states and is associated with adverse clinical outcomes such as panic, anger, and hostility (62). This suggests that individuals with BPD may have deficiencies in their baseline emotional functioning, characterized by heightened emotional intensity and vulnerability. This implies that the primary emotional issues in BPD may not originate from emotional responses but rather from abnormalities in their overall emotional resting state (48). This diminished vagal tone could imply an increased predisposition to fight-or-flight responses, even during resting periods, possibly contributing to the inner tension experienced by individuals with BPD (44). In BPD, symptom severity correlates with reduced heart rate variability (HRV) during rest, linked to heightened susceptibility to negative emotions and adverse clinical outcomes. While BPD individuals exhibit heart rate fluctuations, their HRV remains stable. Additionally, BPD patients often have longer corrected QT dispersion (QTcd) durations, indicating potential cardiac abnormalities or altered autonomic nervous system functioning. They tend to show elevated systolic blood pressure (BPsys), particularly at rest, and consistently heightened diastolic blood pressure (BPdia). Notably, BPD symptom severity doesn’t predict cardiovascular reactivity, indicating consistent differences in their cardiovascular system responses. Varying skin conductance levels (SCL) suggest potential disparities in emotional reactivity. These findings collectively suggest underlying dysregulation in autonomic systems in individuals with BPD, which may contribute to emotional instability and clinical manifestations (44). Furthermore, lower resting scores in left orbicularis oculi electromyography (EMG) in BPD patients may reflect distinct facial muscle activity patterns (42).

### Study limitations

The findings in this review should be considered in light of certain limitations observed in the included articles. Most studies primarily focused on female participants, potentially limiting generalizability, despite more recent research challenging the perception that BPD mainly affects females. Diagnostic bias toward females results from their greater likelihood to seek early mental health assistance, while males may delay diagnosis due to substance abuse and incarceration. Diverse gender representation in BPD research is crucial (63, 64). Additionally, the concentration of studies on young adults aged 25-35 neglects other age groups with BPD. Geographic concentration in Europe could introduce regional biases. Sample sizes varied significantly, some being very small, and some studies lacked comparisons with healthy controls or other groups, making it difficult to draw clear inferences. Nearly half of the studies had comorbidity within their samples, potentially confounding observed cues specific to BPD. Diagnostic heterogeneity arose from varying diagnostic methods. Most studies investigated physiological cues, while only a few explored language use, facial expressions, speech, and non-verbal communication. Our review is limited to English language articles, potentially introducing publication bias. We acknowledge the limitation of not being able to include articles in other languages, which could have offered valuable insights into this subject. Similarly, some of our findings may be biased due to the fact that we included conference proceedings, which are not always refereed, and the fact that we did not assess the methodological quality of the included articles. Mentioned limitations underscore the need for further research addressing these constraints and exploring a broader range of observable cues in diverse BPD populations. Future studies should consider comorbid conditions and the impact of medication on individuals with BPD, as well as recognize diagnostic variations. Addressing these factors can offer a more nuanced and accurate portrayal of BPD, facilitating improved assessment and treatment strategies for this complex disorder.

### Clinical implications

Our in-depth study of BPD characteristics uncovers innovative possibilities for precision psychiatry using digital biomarkers. We identify distinct language patterns in individuals with BPD, emphasizing external viewpoints and an increased use of the future tense. Additionally, specific linguistic characteristics unique to individuals with BPD shed light on their thought processes and emotional experiences, enhancing diagnostic precision. These linguistic cues serve as essential building blocks for the development of precise diagnostic algorithms.

In the realm of nonverbal behaviors, our investigation unveils intriguing insights, including an increased frequency of pauses in BPD individuals’ speech. These findings suggest unique communication tendencies and emotional regulation challenges. Moreover, our research highlights significant correlations between prosody (the rhythm and melody of speech) and facial expressions, providing valuable information about emotional states and interpersonal dynamics. These nonverbal cues enhance our ability to recognize and understand the complexities of the disorder.

The integration of physiological measurements into our study represents a significant advance. By analyzing emotional responses and physiological arousal in individuals with BPD, we gain deeper insights into the psychophysiological aspects of the disorder. This knowledge not only aids in early identification but also opens the door to personalized interventions that address the unique needs of each patient.

Our approach aims to complement existing BPD management methods, ushering in an era of precision and customization in mental healthcare. By incorporating machine learning insights into the diagnostic process, we intend to improve the accuracy of BPD assessments and empower clinicians to make more informed decisions. These groundbreaking tools represent a transformative shift in BPD diagnosis and treatment, offering renewed hope for those dealing with this condition. As our research advances, we anticipate improved diagnostic precision and highly personalized treatment strategies, ultimately creating a brighter future for those facing the challenges of BPD. Digital biomarkers play a central role in this transformative journey.

### Conclusion

In conclusion, this review underscores the pressing need for improved BPD diagnosis and management. The integration of AI and observable cues, including language patterns and nonverbal behaviors, charts a promising course towards enhancing diagnostic precision and personalized treatments for individuals with BPD. These unique cues, harnessed by AI-driven machine learning algorithms, stand as a beacon for the potential transformation of BPD management, facilitating early identification and timely interventions. However, this promising future must be approached with caution, addressing significant ethical concerns such as privacy, data security, and bias. Ensuring transparency and preventing the exacerbation of existing disparities are essential to realizing AI’s transformative potential in mental health care. By balancing innovation with ethical considerations, we can pave the way for more equitable and effective solutions, ultimately improving outcomes and quality of life for those contending with BPD.
